# Expression of the AHPND Toxins PirA*^vp^* and PirB*^vp^* Is Regulated by Components of the *Vibrio parahaemolyticus* Quorum Sensing (QS) System

**DOI:** 10.3390/ijms23052889

**Published:** 2022-03-07

**Authors:** Shin-Jen Lin, Jiun-Yan Huang, Phuoc-Thien Le, Chung-Te Lee, Che-Chang Chang, Yi-Yuan Yang, Emily Chia-Yu Su, Chu-Fang Lo, Hao-Ching Wang

**Affiliations:** 1 International Center for the Scientific Development of Shrimp Aquaculture, National Cheng Kung University, Tainan 701, Taiwan; z10303066@email.ncku.edu.tw (S.-J.L.); jyhuang@mail.ncku.edu.tw (J.-Y.H.); s5890134@gmail.com (C.-T.L.); 2 Graduate Institute of Translational Medicine, College of Medical Science and Technology, Taipei Medical University, Taipei 110, Taiwan; m610109010@tmu.edu.tw (P.-T.L.); ccchang168@tmu.edu.tw (C.-C.C.); 3 Translational Medicine Division, Graduate Institute of Biomedical Informatics, College of Medical Science and Technology, Taipei Medical University, Taipei 110, Taiwan; 4 The PhD Program for Translational Medicine, College of Medical Science and Technology, Taipei Medical University and Academia Sinica, Taipei 115, Taiwan; 5 School of Medical Laboratory Science and Biotechnology, College of Medical Science and Technology, Taipei Medical University, Taipei 110, Taiwan; yangyuan@tmu.edu.tw; 6 Core Laboratory of Antibody Generation and Research, Taipei Medical University, Taipei 110, Taiwan; 7 Graduate Institute of Biomedical Informatics, College of Medical Science and Technology, Taipei Medical University, Taipei 110, Taiwan; emilysu@tmu.edu.tw

**Keywords:** acute hepatopancreatic necrosis disease, Pir toxin, AphB, LuxO, gene regulation, EMSA, shrimp, aquaculture, microbiome

## Abstract

Acute hepatopancreatic necrosis disease (AHPND) in shrimp is caused by *Vibrio* strains that harbor a pVA1-like plasmid containing the *pirA* and *pirB* genes. It is also known that the production of the PirA and PirB proteins, which are the key factors that drive the observed symptoms of AHPND, can be influenced by environmental conditions and that this leads to changes in the virulence of the bacteria. However, to our knowledge, the mechanisms involved in regulating the expression of the *pirA*/*pirB* genes have not previously been investigated. In this study, we show that in the AHPND-causing *Vibrio parahaemolyticus* 3HP strain, the *pirA^vp^* and *pirB^vp^* genes are highly expressed in the early log phase of the growth curve. Subsequently, the expression of the PirA*^vp^* and PirB*^vp^* proteins continues throughout the log phase. When we compared mutant strains with a deletion or substitution in two of the quorum sensing (QS) master regulators, *luxO* and/or *opaR* (*luxO^D47E^*, Δ*opaR*, Δ*luxO,* and Δ*opaR*Δ*luxO*), our results suggested that expression of the *pirA^vp^* and *pirB^vp^* genes was related to the QS system, with *luxO* acting as a negative regulator of *pirA^vp^* and *pirB^vp^* without any mediation by *opaR^vp^*. In the promoter region of the *pirA^vp^*/*pirB^vp^* operon, we also identified a putative consensus binding site for the QS transcriptional regulator AphB. Real-time PCR further showed that *aphB^vp^* was negatively controlled by LuxO*^vp^*, and that its expression paralleled the expression patterns of *pirA^vp^* and *pirB^vp^*. An electrophoretic mobility shift assay (EMSA) showed that AphB*^vp^* could bind to this predicted region, even though another QS transcriptional regulator, AphA*^vp^*, could not. Taken together, these findings suggest that the QS system may regulate *pirA^vp^/pirB^vp^* expression through AphB*^vp^*.

## 1. Introduction

*Vibrio parahaemolyticus* is an opportunistic marine pathogen often found in the ocean and estuary environment [1]. In general, *V. parahaemolyticus* is recognized as an etiologic agent that causes acute gastroenterocolitis and diarrhea after human consumption of contaminated seafood [2,3]. Consequently, most earlier studies were on the pathogenesis of *V. parahaemolyticus* in humans and focused on virulence factors such as its hemolysins, T3SSs, and T6SSs [4,5,6]. However, several strains of *V. parahaemolyticus* were also identified as causative agents of the newly emergent acute hepatopancreatic necrosis disease (AHPND) in shrimp [7]. Since the first outbreak in China in 2009, AHPND has rapidly spread across southeast Asia and reached as far as central and south America, leading to huge losses in the aquaculture industry [8,9,10]. AHPND induces early mortality, usually within 35 to 45 days after stocking post-larvae shrimp in cultivation ponds [11]. Characteristic AHPND symptoms in shrimp include a pale and atrophied hepatopancreas (HP) with an empty stomach and midgut [7,9]. Histological examination has further shown that AHPND causes sloughing of the HP tubule epithelial cells into the HP tubule lumens [7,9], and this has become the main diagnostic criterion to confirm an AHPND infection. AHPND-causing strains harbor a 70-kbp plasmid (pVA1) that produces the “Photorhabdus insect-related” (Pir)-like binary toxins, PirA*^vp^* and PirB*^vp^* [12]. These two AHPND-associated toxins form a complex that is structurally homologous to the insecticidal Cry toxin [12,13,14], and they have been confirmed as the key factors that drive AHPND pathogenesis [12]. However, while previous studies have increased our understanding of the structural and functional characteristics of PirA*^vp^* and PirB*^vp^*, the mechanism by which the *pirA^vp^* and *pirB^vp^* genes are regulated still remains unknown. Here, we investigate this mechanism and show that the quorum sensing (QS) system may play an important role.

QS is a cell density-dependent process that regulates the expression of a number of genes in both Gram-positive and Gram-negative bacteria [15]. The QS system achieves this regulation by a series of control factors, including LuxO, OpaR (a homolog of LuxR), and AphA [16,17]. QS-regulated genes are involved in many important physiological activities, such as biofilm formation, bioluminescence, virulence factor production, conjugation, plasmid transfer, antibiotic production, cell mobility, and sporulation [15]. The importance of the QS system in AHPND pathogenicity was also recently demonstrated: extract from *V. alginolyticus* BC25 contained the anti-QS compounds Cyclo-(L-Leu-L-Pro) and Cyclo-(L-Phe-L-Pro), both of which had anti-QS activity, and pre-treatment with *V. alginolyticus* BC25 reduced mortality after challenge with the AHPND-causing strain *V. parahaemolyticus* PSU5591 [18]. However, this study did not investigate the mechanism by which the QS system regulates the virulence of AHPND-causing *V. parahaemolyticus*.

Here we show how LuxO*^vp^*, which is an important regulator of QS in *Vibrio* spp., affects the gene expression of the key AHPND pathogenic factors *pirA^vp^* and *pirB^vp^*. First, by monitoring the gene/protein expression of PirA*^vp^* and PirB*^vp^* during the growth of *V. parahaemolyticus*, we found the appearance of these two toxins is cell density-dependent. We then confirmed that, at the low cell density stage (LCD, OD_600_ ≈ 0.6), the deletion of LuxO*^vp^* significantly increased the gene/protein expression of PirA*^vp^* and PirB*^vp^* in *V. parahaemolyticus*. Next, in a PirA*^vp^*/PirB*^vp^* operon analysis, a possible DNA binding site for the *Vibrio* regulator AphB was identified. AphB is known as an activator, which plays a central role in virulence gene expression in both *Vibrio cholerae* and *Vibrio alginolyticus* [19,20]. It also plays a regulatory role as a QS control factor of LuxR [20]. Here, using EMSA (electrophoretic mobility shift assay), we confirmed that *V. parahaemolyticus* AphB (AphB*^vp^*) bound to the proposed promoter region of the PirA*^vp^*/PirB*^vp^* operon. Using real-time polymerase chain reactions, we also found a correlation between the expression levels of LuxO*^vp^* and AphB*^vp^*. At low cell density, the expression of AphB*^vp^* was increased by 1.7 fold in LuxO*^vp^*-deleted *V. parahaemolyticus*, and this increase was positively correlated to the gene/protein expression of PirA*^vp^* and PirB*^vp^* under the same conditions. Taken together, our results show that the *pirA^vp^*/*pirB^vp^* genes are regulated by components of the QS system, particularly by AphB*^vp^*.

This is the first report to investigate the influence of the bacterial physiological system on the *pirA^vp^*/*pirB^vp^* genes. Our findings will be helpful for the development of APHND prevention and/or control strategies in the future.

## 2. Results

### 2.1. The Expression Levels of pirA^vp^/pirB^vp^ during Different Growth Phases of V. parahaemolyticus

**Figure 1:**
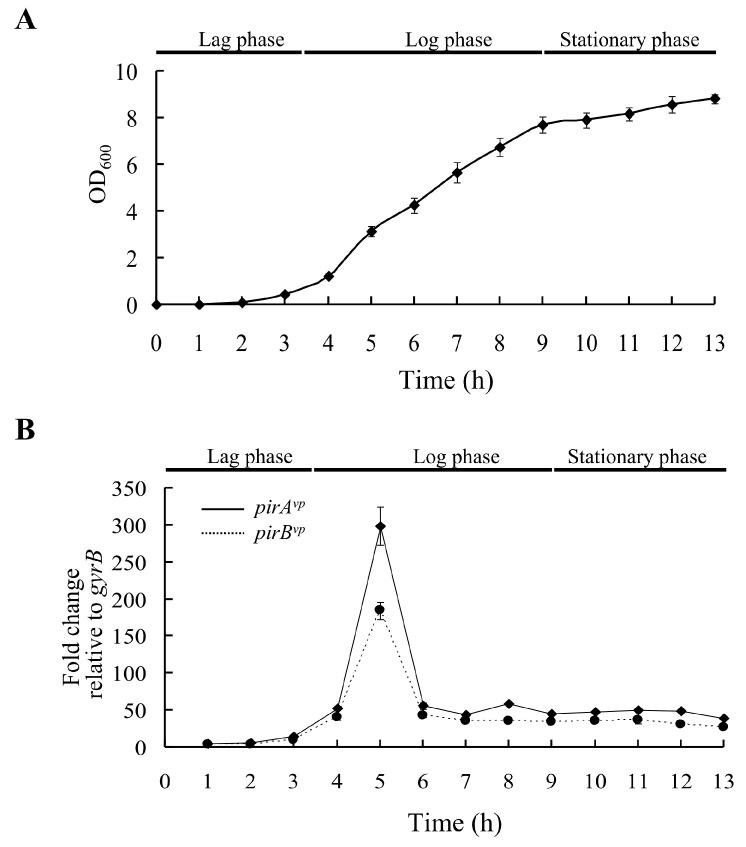
Growth curve and *pirA^vp^* and *pirB^vp^* expression levels in *V. parahaemolyticus* strain 3HP. (**A**) *V. parahaemolyticus* strain 3HP was cultured in LB (2% NaCl), and the growth curve was recorded every hour until 13 h after inoculation. (**B**) Relative mRNA expression of *pirA^vp^* and *pirB^vp^* in the strain 3HP during different growth phases. Total RNA was extracted from *V. parahaemolyticus* 3HP collected at the indicated time points (OD_600_ 0.01–8.82), and analyzed by real-time qPCR with specific primers for *pirA^vp^* and *pirB^vp^*, respectively. Expression levels are shown relative to those of the *gyrB* reference gene.

**Figure 2:**
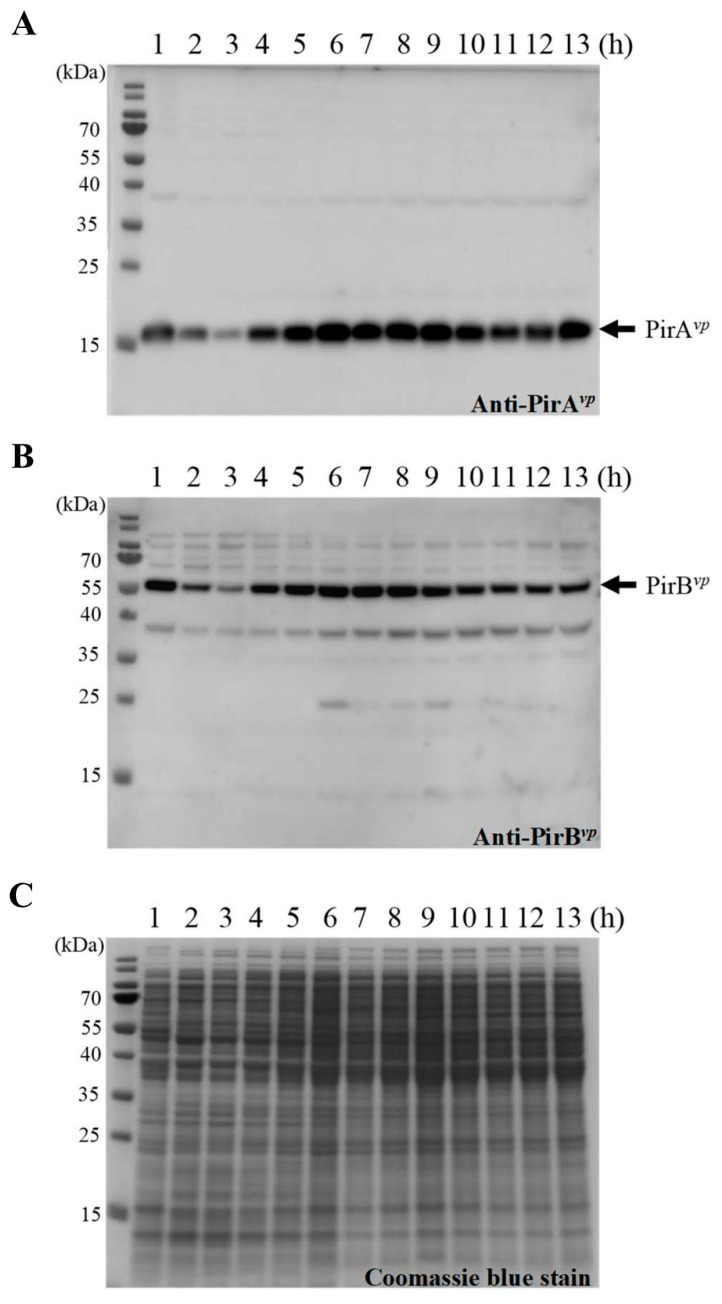
Protein expression levels of endogenous PirA*^vp^* and PirB*^vp^* in *V. parahaemolyticus* wild type strain (3HP) during different growth phases. Cell lysate (2 μg) was separated, transferred onto a PVDF membrane, and reacted with (**A**) chicken anti-PirA*^vp^* and (**B**) anti-PirB*^vp^* polyclonal antibodies to recognize the endogenous PirA*^vp^* and PirB*^vp^*, respectively. (**C**) Loading control (8 μg/lane).

In *Vibrio*, since QS is involved in the regulation of many physiological processes, including several virulence-related systems, we first wanted to determine if the expression level of *pirA^vp^*/*pirB^vp^* was cell density-dependent. We, therefore, recorded the growth curve of the wild type *V. parahaemolyticus*, 3HP strain, and analyzed the gene expression patterns of *pirA^vp^* and *pirB^vp^*. As shown in Figure 1A, the lag phase lasted until about 3.5 h (OD_600_ from 0.01 to ~0.6), the log phase ran until 9 h (OD_600_ from 0.6 to ~7.7), and the stationary phase ran from 9~13 h (OD_600_ from 7.7 to ~8.8). The expression of *pirA^vp^* and *pirB^vp^* remained low until the curve entered the log phase (Figure 1B). Expression levels reached their peaks at 5 h, then declined again at 6 h and continued to remain low (Figure 1B). To determine the protein expression patterns, we used specific anti-PirA*^vp^* and anti-PirB*^vp^* antibodies to detect the PirA*^vp^* and PirB*^vp^* proteins at each time point. As shown in Figure 2, the PirA*^vp^* and PirB*^vp^* expression levels were low in the lag phase except for 1 h. The over-presence at this time point might be due to carry-over from 15 h culture prior to inoculation. The expression levels were high in the log phase through to the early part of the stationary phase (4~10 h), after which they returned to lower levels until the end of the recording.

### 2.2. LuxO Is a Negative Regulator for the Expression of pirA^vp^ and pirB^vp^

**Figure 3:**
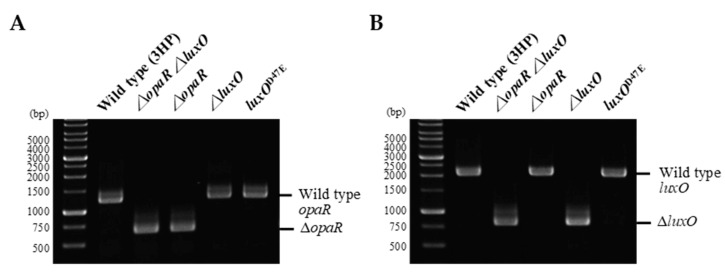
Confirmation of Δ*opaR*, Δ*luxO*, Δ*opaR*Δ*luxO*, and *luxO^D47E^* mutants by PCR. DNAs from the wild-type strain and the mutants were analyzed by PCR with the primers specific to *opaR* (**A**) and *luxO* (**B**). The bands corresponding to the wild type *opaR* and *luxO*, and the deleted *opaR* and *luxO* (Δ*opaR* and Δ*luxO*, respectively) are indicated.

**Figure 4:**
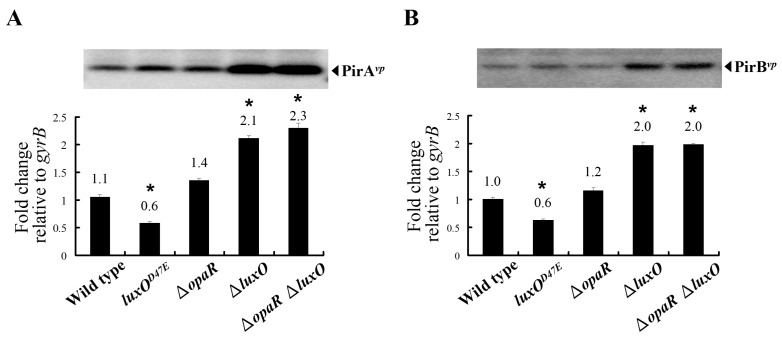
The gene and protein expression levels of (**A**) *pirA^vp^* and (**B**) *pirB^vp^* in the wild type strain (3HP) compared to those in the *luxO^D47E^*, ∆*opaR*, ∆*luxO,* and ∆*opaR*∆*luxO* mutants. Soluble proteins were extracted from cells collected at OD_600_~0.6 and analyzed by immunoblotting with anti-PirA*^vp^* or anti-PirB*^vp^* antibodies (upper panels). Total RNA was extracted from the same batch of bacterial samples, and real-time RT-PCR was carried out using specific primer sets for *pirA^vp^* and *pirB^vp^* (lower panels), respectively. The housekeeping gene, *gyrB* served as an internal control. *: *p* < 0.05.

To better understand whether QS regulation is involved in the expression of *pirA^vp^* and *pirB^vp^*, isogenic mutants of *opaR* and *luxO* were derived from the AHPND-causing strain 3HP. These mutants had a deletion in *opaR* (Δ*opaR*) or *luxO* (Δ*luxO*), or in both *opaR* and *luxO* (Δ*opaR*Δ*luxO*), and they were constructed by allelic gene exchange as described previously [21]. Successful deletion in the different mutants was confirmed by using *opaR*-specific and *luxO*-specific primer sets (Figure 3). In addition, we also mutated the 47th amino acid of LuxO*^vp^* from aspartic acid (D) to glutamic acid (E) to mimic the permanently active form of LuxO*^vp^* (*luxO^D47E^*). All mutants exhibited similar growth rates to that of the wild-type strain in LB+ medium (Figure S1). As shown in Figure 4, the gene expression levels of *pirA^vp^* and *pirB^vp^* were down-regulated to about 60% when the active form of LuxO*^vp^* was mimicked, while the expression levels were up-regulated by about 2 folds with the *luxO^vp^* deletion mutants (i.e., Δ*luxO* and Δ*opaR*Δ*luxO*). By contrast, the *opaR^vp^* deletion mutant had only a small, statistically insignificant effect on the expression of *pirA^vp^* and *pirB^vp^*, suggesting that although *opaR^vp^* is one of the core regulators of the QS system, unlike *luxO^vp^*, it plays only a minor role in the expression of *pirA^vp^* and *pirB^vp^*. Similar effects were also seen in the protein expression levels of PirA*^vp^* and PirB*^vp^*, although we note that, despite the reduction in mRNA expression, protein levels were not reduced by the *luxO^D47E^* mimic (Figure 4A,B, upper panels). Taken together, these results suggest that the expression of *pirA^vp^* and *pirB^vp^* is negatively regulated by *luxO^vp^*, but not by *opaR^vp^*.

### 2.3. The QS Transcription Factor AphB^vp^ Is a Possible Regulator for pirA^vp^ and pirB^vp^

**Figure 5:**
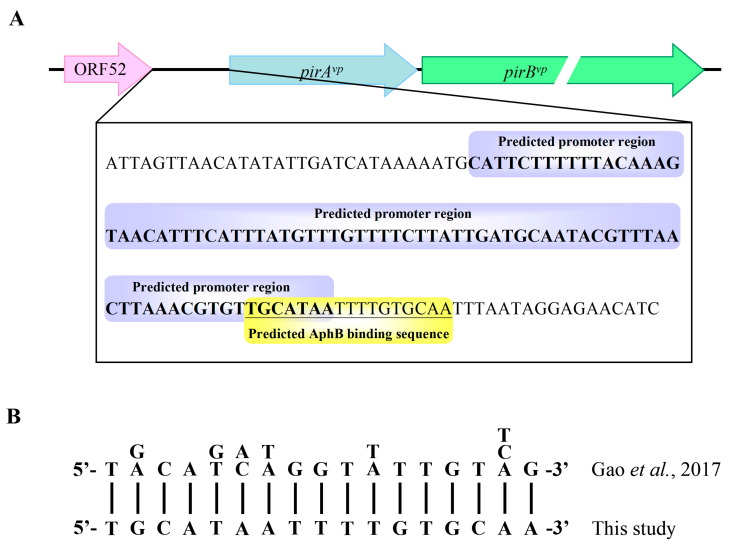
Schematic representation of the predicted AphB binding site in the *pirA^vp^/pirB^vp^* promoter region. (**A**) The predicted *pirA^vp^*/*pirB^vp^* promoter region is bolded and shaded blue. The putative AphB binding sequence, 5′-TGCATAATTTTGTGCAA-3′, is underlined and shaded yellow. (**B**) Alignment of the AphB binding sequence on the *V. alginolyticus* chromosome [20] with the predicted AphB*^vp^* binding sequence in the predicted *pirA^vp^*/*pirB^vp^* promoter region (this study).

**Figure 6:**
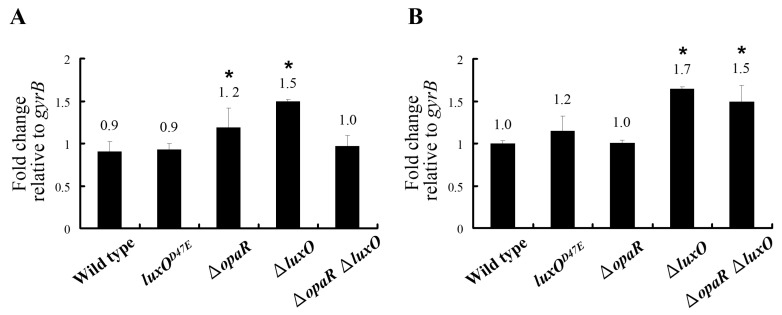
The gene expression levels of (**A**) *aphA^vp^* (**B**) *aphB^vp^* in the wild type strain (3HP), *luxO^D47E^*, ∆*opaR*, ∆*luxO,* and ∆*opaR*∆*luxO* mutants at OD_600_~0.6. The expression pattern of *aphB^vp^* was similar to the expression patterns of *pirA^vp^* and *pirB^vp^*. By contrast, the expression pattern of *aphA^vp^* was not a good match. The housekeeping gene, *gyrB* served as an internal control. *: *p* < 0.05.

Since the key QS transcription factor, OpaR is evidently not involved in the regulation of *pirA^vp^* and *pirB^vp^*, we further analyzed the predicted promoter region of the *pirA^vp^/pirB^vp^* operon, and found that there was a sequence (5′-TGCATAATTTTGTGCAA-3′), which was similar to the consensus sequence of the AphB binding site, 5′-T-G/A-C-A-G/T-A/C-T/A-G-G-T-T/A-T-T-G-T-T/C/A-G-3′ [20] (Figure 5). Using real-time PCR, we found that for *aphB^vp^*, the elevated gene expression levels of the *luxO^vp^* deletion mutants Δ*luxO* and Δ*opaR*Δ*luxO* were similar to those seen for *pirA^vp^* and *pirB^vp^* (Figure 6B). Conversely, the expression levels of another important QS transcription factor, *aphA^vp^*, did not correspond so closely to the expression patterns of *pirA^vp^* and *pirB^vp^* (Figure 6A). Taken together, these results suggest that AphB*^vp^*, but not AphA*^vp^*, may be important for the expression of *pirA^vp^* and *pirB^vp^*.

### 2.4. His-AphB^vp^ Binds with the Predicted Promoter Region of pirA^vp^/pirB^vp^

**Figure 7:**
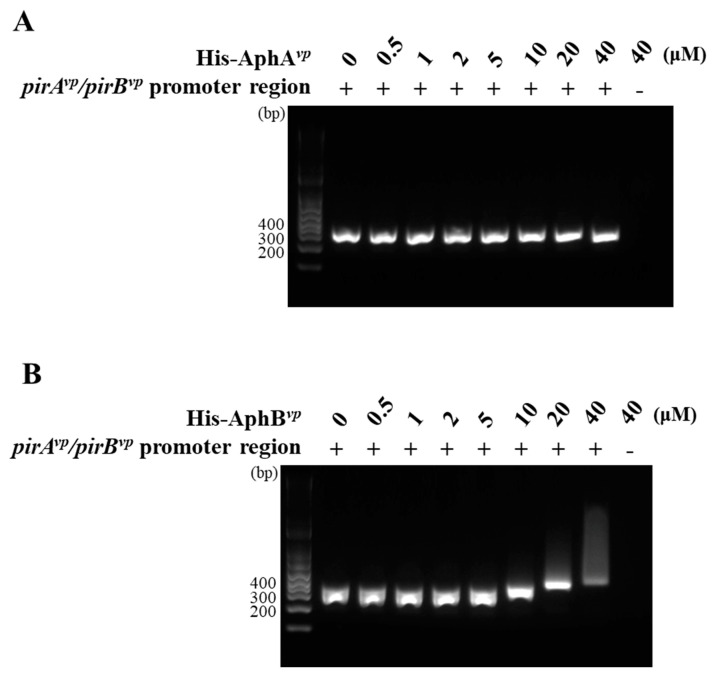
AphB*^vp^* binds to the predicted *pirA^vp^*/*pirB^vp^* promoter region in an electrophoretic mobility shift assay (EMSA). (**A**) As the concentration of His-AphA*^vp^* increased, the DNA fragments produced by PCR amplification of the predicted promoter region remained unshifted. (**B**) By contrast, an upward shift was seen for His-AphB*^vp^* at concentrations of 10 μM and above.

In order to verify whether AphB*^vp^* could bind directly to the predicted AphB*^vp^* binding site located upstream of *pirA^vp^*/*pirB^vp^*, we amplified the fragment 300 bp upstream of the *pirA^vp^*/*pirB^vp^* operon by PCR, and mixed it with the indicated concentrations (0, 0.5, 1, 2, 5, 10, 20, and 40 μM) of the recombinant His-tagged Aph proteins His-AphB*^vp^* and His-AphA*^vp^*. As shown in Figure 7B, the DNA fragments shifted upward at His-AphB*^vp^* concentrations of 10 μM and above, showing that His-AphB*^vp^* was able to bind to this DNA fragment. By contrast, no DNA band shift was seen for His-AphA*^vp^* (Figure 7A), suggesting that binding to the predicted promoter region of *pirA^vp^*/*pirB^vp^* is His-AphB*^vp^*-specific.

**Figure 8:**
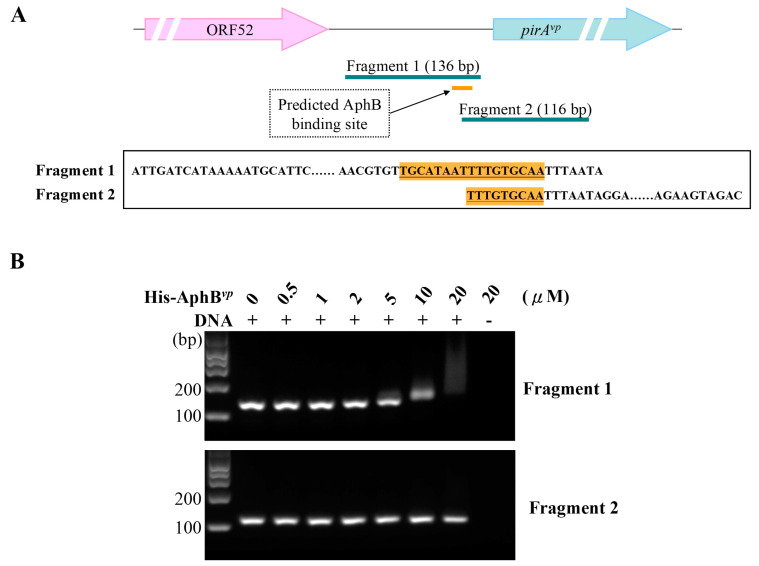
The predicted AphB*^vp^* binding sequence is important for binding AphB*^vp^*. (**A**) The *pirA^vp^*/*pirB^vp^* promoter region was truncated into fragments that included either the full (Fragment 1) or partial (Fragment 2) predicted AphB*^vp^* binding sequence. The head-tail sequences of these fragments are shown in the box, and the nucleotides in the predicted AphB*^vp^* binding sequence are shaded orange. (**B**) The EMSA results show that AphB*^vp^* bound only to the full binding sequence (Fragment 1).

To further verify the importance of the putative AphB binding sequence in the predicted promoter region of *pirA^vp^*/*pirB^vp^*, we truncated the DNA fragment into Fragment 1 (136 bp), which contained the complete AphB binding sequence, and Fragment 2 (116 bp), which contained only half of the AphB binding sequence (Figure 8A). After incubation with the indicated concentrations of His-AphB*^vp^*, we observed shifting with Fragment 1 at concentrations of 5~10 μM and above, whereas there were no shifts observed at any concentration with Fragment 2 (Figure 8B). These results confirmed the importance of the predicted sequence for AphB*^vp^* binding and thus for its potential role in the regulation of *pirA^vp^* and *pirB^vp^* expression.

## 3. Discussion

PirA*^vp^* and PirB*^vp^* have been confirmed as critical pathogenic factors of AHPND [12]. In a previous study, we further showed that PirA*^vp^* and PirB*^vp^* formed a heterotetramer in solution, and based on this tetramer’s structural similarity to the Cry toxin, we proposed that the binary toxin may destroy host cells by means of a mechanism similar to that used by Cry [14]. In particular, we suggested that the role of the PirA*^vp^* component is to recognize the glycan of its receptor on the host cells, after which PirB*^vp^* penetrates the cell membrane to form an unregulated channel, ultimately leading to critical cell damage [14]. Other reports have further shown that both PirA*^vp^* and PirB*^vp^* can bind directly to the receptor *Lv*APN1, and that PirB*^vp^* was translocated to the cytoplasm and nucleus of hemocytes [22,23], suggesting that the PirA*^vp^* and PirB*^vp^* toxins might be involved in other, additional pathogenic mechanisms. Until the present study, however, the mechanisms that might regulate expression of the *pirA* and *pirB* genes themselves had remained unknown. Here, we found a relationship between cell density and high expression levels of *pirA^vp^* and *pirB^vp^* in the early log phase (Figure 1). In addition, starting at around the same time as the peak of the gene expression levels, we also observed sustained elevated levels of PirA*^vp^* and PirB*^vp^* protein throughout the entire log phase and on into the stationary phase (Figure 1 and Figure 2).

It is already known that QS system is involved in the regulation of many virulence factors in *Vibrio*, including the genes of the type-III secretion systems (T3SS1 and T3SS2), type-VI secretion systems (T6SS1 and T6SS2), and the thermostable direct hemolysin genes *tdh1* and *tdh2* [24,25,26]. Here, by using strains with mutations in key molecules of the QS system, we found that the expression of *pirA^vp^* and *pirB^vp^* was also regulated by components of the QS system and that it was negatively regulated by LuxO*^vp^* (Figure 4).

**Figure 9:**
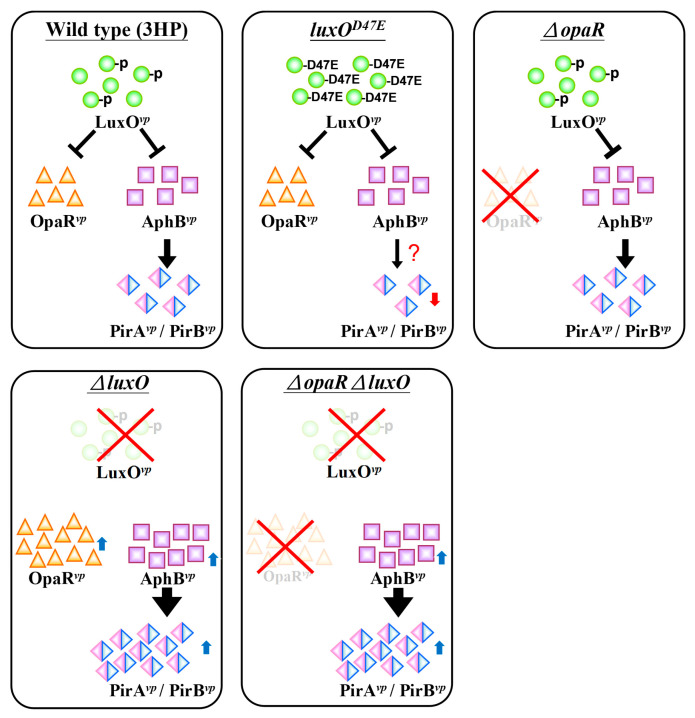
The relationships between LuxO*^vp^*, AphB*^vp^* and PirA*^vp^*/PirB*^vp^* at OD_600_~0.6. In the first three strains (i.e., wild type strain 3HP, the activated mimic strain *luxO^D47E^*, and the OpaR deletion strain Δ*opaR*), the phosphorylated LuxO*^vp^* acted to limit the free expression of AphB*^vp^* and its downstream genes, *pirA^vp^*/*pirB^vp^*. Conversely, when LuxO was deleted (strains Δ*luxO* and Δ*opaR*Δ*luxO*)*,* AphB*^vp^* was significantly up-regulated, and this further promoted the expression of PirA*^vp^*/PirB*^vp^*.

Although the upstream region of the *pirA^vp^*/*pirB^vp^* operon did not include a consensus LuxO binding sequence (5′-TTGCAW_3_TGCAA-3′, where W stands for A or T; [27]), we found a possible AphB binding site as shown in Figure 5. In addition, we also found that the expression of *aphB^vp^* was affected by mutation of the QS component *luxO^vp^* (Figure 6). The upregulated expression pattern of *aphB^vp^* was similar to the pattern seen for *pirA^vp^* and *pirB^vp^* under the same conditions (Figure 4). The possible AphB binding sequence we observed (5′-TGCATAATTTTGTGCAA-3′) diverges from other established binding motifs, such as those of *tcpP* (5′-TGCAAN_7_TTGCA-3′), *toxR* (5′-TGCAAN_7_ATGGA-3′), *aphB* (5′-TGCAAN_7_TGTCA-3′), and a consensus sequence (5′-TGCAGN_7_TGTTG-3′), as well as *cadC* I (5′-TTAAAN_7_ACTTA-3′) and *cadC* II (5′-TACGTN_7_GGCTA-3′) [20]. Nevertheless, despite this high divergence, our EMSA results showed that the binding between AphB*^vp^* and the predicted AphB binding site was both direct and specific (Figure 7 and Figure 8). AphB*^vp^* (but not AphA*^vp^*) thus appears to act as an enhancer of *pirA^vp^* and *pirB^vp^*. Given AphB*^vp^*’s role as a regulator of QS, we hypothesize that the QS system regulates the expression of *pirA^vp^* and *pirB^vp^* via AphB*^vp^* (Figure 9, upper left panel). We also found that *pirA^vp^* and *pirB^vp^* were down-regulated by the permanently active *luxO^vp^* mutant even though there was no significant change in *aphB^vp^*. This suggests that the regulation of the PirA*^vp^*/PirB*^vp^* genes may not be controlled only by AphB*^vp^,* but that other LuxO*^vp^*-related effectors may also be involved (Figure 9, upper center panel). The proposed regulatory mechanisms and outcomes for LuxO*^vp^*, AphB*^vp^*, and PirA*^vp^*/PirB*^vp^* in the wild type (3HP) and mutant strains (*luxO^D47E^*, Δ*opaR*, Δ*luxO,* and Δ*opaR*Δ*luxO*) are shown in Figure 9.

While AphA is a well-studied QS regulator [28,29], there are relatively few studies on AphB. These studies include its regulation of virulence [19,20] and the role it plays in survival under particular conditions [30]. AphB is also a positive regulator of LuxR/OpaR activity, and it activates the expression of the exotoxin Asp [20]. These studies, together with the recent finding that anti-QS compounds may reduce AHPND pathogenicity [18], all suggest that the QS system might be critically important for regulating the virulence of AHPND-causing bacteria.

To this body of evidence, we now add the results of the present study, which suggests that the QS system might be modulating virulence by regulating expression of the *pirA^vp^/pirB^vp^* genes through AphB*^vp^*. This new insight into the pathogenic mechanisms of AHPND points toward the QS system as a possible target for therapeutics that might one day be able to control the virulence of AHPND-causing bacteria and prevent AHPND.

## 4. Materials and Methods

### 4.1. Growth Curve Measurement and Sample Collection

All *V. parahaemolyticus* strains (3HP, LuxO^D47E^, Δ*opaR*, Δ*luxO,* and Δ*opaR* Δ*luxO*) were activated by culturing on LB+ agar plates (that is, LB agar plates that contained 2% NaCl) at 30 °C for 16 h. From these plates, 3 single colonies were transferred into 5 mL LB+ medium (LB medium that contained 2% NaCl) and incubated at 30 °C for 3 h to OD_600_~1.0. The culture was then diluted 100-fold, transferred into 50 mL fresh LB+ medium and cultured at 30 °C for 15 h with shaking at 200 rpm. Dilutions (1:1000) of these overnight cultures were further sub-cultured into 500 mL LB+ medium in a 2L flask, incubated at 30 °C with shaking at 200 rpm, and the OD_600_ was measured every hour for a total of 13 h. At the same time, 3HP cells were collected from part of the culture every hour, and temporarily stored at −80 °C prior to subsequent RNA and protein extractions. Other batches of sample cells of 3HP, LuxO^D47E^, Δ*opaR*, Δ*luxO,* and Δ*opaR* Δ*luxO* were collected at low cell density (LCD; OD_600_~0.6) and temporarily stored at −80 °C for later use.

### 4.2. RNA Extraction and Real-Time PCR

Total bacterial RNA was extracted using RareRNA reagent (Blossom Biotechnologies, Inc.; Taipei, ROC). DNaseI (Invitrogen) was used to digest any DNA contamination. From 1 µg of total RNA, cDNA was synthesized by using M-MLV reverse transcriptase (Promega; Madison, WI, USA) with random hexamers. Using specific primers (Table 1), real-time PCR was carried out to quantify the expression levels of target genes (*pirA^vp^*, *pirB^vp^*, *aphA^vp^*, and *aphB^vp^*) and an internal control gene (*gryB^vp^*, a housekeeping gene). The reaction mixture contained 1 μL cDNA, 0.2 μM forward primer, 0.2 μM reverse primer, 1× ChamQ Universal SYBR qPCR Master Mix (Vazyme Biotech Co. Ltd.; Nanjing, China) in a total volume of 20 μL, and thermal cycling was performed using a LightCycler^®^ 96 System (Roche; Basel, Schweiz) as follows: 95 °C for 2 min followed by 40 cycles of 95 °C for 10 s and 60 °C for 30 s, and an extension cycle of 95 °C for 10 s, 65 °C for 60 s and 97 °C for 60 s.

To calculate Δ*C_t_*, the threshold cycle values of the internal control gene (*C_t_*_gyrB_) were first subtracted from the threshold cycle values of the target genes. Next, either the initial Δ*C_t_* for the “1-h” timepoint was subtracted from the Δ*C_t_*s of the other timepoints (i.e., 2–13 h), or else the Δ*C_t_* for the wild-type 3HP strain was subtracted from the Δ*C_t_* of all the groups (3HP, *luxO^D47E^*, Δ*opaR*, Δ*luxO,* and Δ*opaR* Δ*luxO*) to obtain ΔΔ*Ct* values. The fold change of the gene expression levels was then expressed as 2^−ΔΔ*Ct*^ [31], and the data presented as mean ± SD. Statistically significant differences were tested using an unpaired Student’s *t*-test (*p* < 0.05).

### 4.3. Protein Extraction and Western Blots

Two batches of bacterial samples were removed from storage at −80 °C and lysed immediately using B-PER™ Bacterial Protein Extraction Reagent (ThermoFisher Scientific; Waltham, MA, USA) containing 1 mM PMSF, 1 mM EDTA, and 120 μg/mL DNaseI. The suspensions were inverted at room temperature for 10 min, and the cell debris was removed by centrifuging at 13,000× *g* for 10 min. Two micrograms of cell lysates were separated by 12.5% SDS-PAGE, transferred onto a PVDF membrane, and blocked with 5% skim milk at 4 °C overnight. The blots were then hybridized with chicken anti-PirA*^vp^* or chicken anti-PirB*^vp^* polyclonal antibodies (1:5000 diluted with 5% skim milk). After 1 hour of incubation at room temperature, the blots were washed 3 times with PBST solution (1× PBS contained 0.1% Tween-20). The blots were further incubated with donkey anti-chicken-HRP conjugated secondary antibody (Jackson; West Grove, PA; 1:10,000 diluted with 5% skim milk) at room temperature for 1 h. Following 3 more washes, the protein bands were visualized using a chemiluminescence reagent (GE Healthcare; Chicago, IL, USA) and detected with an Amersham Imager 600 (GE Healthcare; Chicago, IL, USA). For loading controls, 8 μg of cell lysates were separated with another 12.5% SDS-PAGE, and stained with Coomassie blue.

### 4.4. Construction of the ΔopaR, ΔluxO, ΔopaRΔluxO, and luxO^D47E^ Mutants

The in-frame Δ*opaR*, Δ*luxO*, and Δ*opaR* Δ*luxO* mutants were constructed by in vivo allelic exchange as described previously [21]. Briefly, DNA fragments from the down- and up-stream regions of *opaR* and *luxO* were amplified, respectively, with the primer sets opaR-1/opaR-2 and opaR-3/opaR-4, and luxO-1/luxO-2 and luxO-3/luxO-4 (Table 1). These fragments were cloned into pGEM-T^®^ Easy vector (Promega; Madison, WI, USA) in the correct orientation to generate a recombinant fragment containing a 627 bp- and a 1323 bp-deletion in *opaR* and *luxO*, respectively. The DNA fragments were removed from the pGEMT^®^-easy vector by enzyme digestion with *Sac*I and *Sal*I, respectively, and then cloned into the suicide vector pDS132. The suicide plasmids containing either the Δ*opaR* or Δ*luxO* fragment were transformed into *Escherichia coli* S17-1λ*pir* [21], and then transferred into *V. parahaemolyticus* 3HP by conjugation to facilitate allelic exchange to produce the mutants. The double mutant was obtained by introducing the Δ*luxO* fragment into an already-constructed Δ*opaR* mutant by the method described above. For the *luxO^D47E^* mutant, a 2340-bp fragment amplified by the primers luxO-1 and luxO-16 was given a single nucleotide mutation (GAT to GAG) using a QuikChange^®^ Site-Directed Mutagenesis Kit (Stratagene; La Jolla, CA, USA). This *luxO^D47E^*-containing fragment was introduced into *V. parahaemolyticus* 3HP by allelic exchange to generate the mutant.

### 4.5. Confirmation of the ΔopaR, luxO, ΔopaRΔluxO and luxO^D47E^ Mutants by PCR

Genomic DNA was extracted from 3HP and the mutants using a Genomic DNA Extraction Kit (Bioman; New Taipei City, ROC) according to the manufacturer’s instructions. The *opaR* and *luxO* genes from all of the isolated mutants were then checked by PCR with the *opaR-* and *luxO-*specific primers opaR-5/opaR-6 and luxO-5/luxO-6 (Table 1), respectively. The DNA sequences were also determined to confirm that the deletion was in-frame, or, in the case of *luxO^D47E^,* that the single nucleotide mutation was correct.

### 4.6. Plasmid Construction for Recombinant Protein Expression

The codons in the coding sequences of *aphA^vp^* (CP045794; region 937101-937640) and *aphB^vp^* (WP069541384) were optimized, synthesized, and cloned into pET21b vector (Novagen; Madison, WI, USA) by GenScript Inc. (Piscataway, NJ, USA). Using the resulting plasmids as templates, the *aphA^vp^* and *aphB^vp^* genes were amplified with the primer sets AphA-F-*Nde*I/AphA-R-*Xho*I and AphB-F-*Nde*I/AphB-R-*Xho*I (Table 1), respectively, and then subcloned into pET28a vector (Novagen; Madison, WI, USA). The resulting plasmids were named *aphA^vp^*-pET28a and *aphB^vp^*-pET28a, respectively.

### 4.7. Expression and Purification of Recombinant His-AphA and His-AphB

To express the recombinant AphA*^vp^* and AphB*^vp^*, the *aphA^vp^*-pET28a and *aphB^vp^*-pET28a plasmids were, respectively, transformed into *E. coli* strain BL21 (DE3) cells. For AphA*^vp^*, the transformed cells were inoculated into 50 mL of fresh LB medium and grown at 37 °C for 12–14 h. Three ml of this overnight culture was then added to 500 mL of fresh LB medium in a 2L flask, and grown at 37 °C until the OD_600_ of the culture reached 0.4. IPTG was then added to a final concentration of 0.4 mM, and the culture was incubated at 16 °C for 20 h. The cells were then collected, resuspended in binding buffer (20 mM Tris-base, 500 mM NaCl, 20 mM imidazole, pH 8.0) containing 1 mM PMSF, 100 μg/mL lysozyme and 10 μg/mL DNase I, and homogenized by sonication on ice. After the cell debris was removed by centrifugation, the supernatant was filtrated using a 0.45 μm filter and loaded onto a 5 mL HisTrap HP column (GE Healthcare; Chicago, IL, USA). The column was washed with 100 mL of binding buffer and then eluted with a 20–500 mM imidazole gradient. The eluted recombinant protein was concentrated and loaded onto a Superdex 75 gel filtration column (GE Healthcare; Chicago, IL, USA) using 20 mM Tris-base, 500 mM NaCl, pH 8.0 as a running buffer. The protein concentration was measured by the Bradford method. For AphB*^vp^*, all of the culture and purification processes were the same as for AphA*^vp^*, except that for the subculture step, 10 mL of the overnight culture was inoculated into the 500 mL of fresh LB+ medium.

### 4.8. Electrophoretic Mobility Shift Assay (EMSA)

ProOpDB (Prokaryotic Operon DataBase) was used to predict possible promoter sequences in the upstream region of the *pirA^vp^/pirB^vp^* operon [32]. The DNA fragment that contained this predicted promoter region was further amplified by PCR using the primer set *pirAB* promoter-F1-*Nde*I/*pirAB* promoter-R1-*Xho*I, *pirAB* promoter-F2-*Nde*I/*pirAB* promoter-R2-*Xho*I (for Fragment 1, which included the complete predicted AphB binding sequence) or *pirAB* promoter-F3/*pirAB* promoter-R3-*Xho*I (for Fragment 2, which included only a partial predicted AphB binding sequence) (Table 1). For EMSA, recombinant His-AphA or His-AphB was mixed with the DNA fragment in a reaction buffer (20 mM Tris, pH 8.0, 100 mM NaCl) to final concentrations of 0, 0.5, 1, 2, 5, 10, 20, 40 µM (proteins), and 135 nM (DNA), and incubated at 25 °C for 20 min. The reactants were analyzed with 2% agarose gels, and stained by SYBR^®^ Green I nucleic acid gel stain (Sigma-Aldrich; Burlington, MA, USA).

## Figures and Tables

**Table 1:** Primers used in this study.

Primer Name	Primer Sequence (5′–3′)	Usage
opaR-1	GAGACCGTTGAAGCATCG	Mutant construction
opaR-2	CAGGTACCGAGTCCATATCCATTT	Mutant construction
opaR-3	CAGGTACCCGAACACTAAAGCTCA	Mutant construction
opaR-4	CAGAGCTCGGGTACGGTTTACCAC	Mutant construction
opaR-5	GTTCTAGAGTGGGTTGAGGTAGGT	Mutant selection
opaR-6	GGTCTAGAGTTGGTACTAACGGTG	Mutant selection
luxO-1	GAGAGCTCCGTATTCGTGCCGCCAAAG	Mutant construction
luxO-2	CTGGTACCGCTGTATCCTCAACCATC	Mutant construction
luxO-3	GAGGTACCAGAAGAGCGGCAGAAGGTG	Mutant construction
luxO-4	CTGGTACCCGACCGCTGGATGCAATC	Mutant construction
luxO-5	GCTCTAGACGGCTGAGAAGCGTGATG	Mutant selection
luxO-6	GGTCTAGAGAGTCCAAGAGCGATACG	Mutant selection
pirAQF	TTAGCCACTTTCCAGCCGC	qPCR
pirAQR	CCGGAAGTCGGTCGTAGTGT	qPCR
pirBQF	TCGTTATCAGCCCACGCAG	qPCR
pirBQR	TTTCACCGATTCTGATGTGCA	qPCR
aphAQF	GAAACTTATGGCTTGTGCTG	qPCR
aphAQR	GCGGCTTCAATTTCTTTGTA	qPCR
aphBQF	TGGGATGTTATTTTCCGTGT	qPCR
aphBQR	CTGCTAGATAGTCTTGGCTG	qPCR
gyrB-1	GAAGGTGGTATTCAAGCGTTCG	qPCR
gyrB-2	GAGATGCCGTCTTCACGTTCT	qPCR
AphA-F-*Nde*I	AATGCCCCATATGAGCCTGCCGCACGTG	Protein expression
AphA-R-*Xho*I	CCGCTCGAGTTAGCCAATAACTTCCAGCTCG	Protein expression
AphB-F-*Nde*I	AAGGCCCCATATGAAGCTGGACGATCTGAACC	Protein expression
AphB-R-*Xho*I	GCCGCTCGAGTTAGTGGATGTTATACGCAATAACAAAG	Protein expression
*pirAB* promoter-F1-*Nde*I	AGGCTTCCATATGAGTGGAAATGGTGAACTTGCGGAAG	EMSA
*pirAB* promoter-R1-*Xho*I	AAGCTCGAGGTCTACTTCTGTGACGCCTCCG	EMSA
*pirAB* promoter-F2-*Nde*I	AGGCTTCCATATGATTGATCATAAAAATGCATTCTTTTTTACAAAG	EMSA
*pirAB* promoter-R3-*Xho*I	AAGCTCGAGTATTAAATTGCACAAAATTATGCAACACG	EMSA
*pirAB* promoter-F7	TTTGTGCAATTTAATAGGAGAACATCATGAG	EMSA
*pirAB* promoter-R-*Xho*I	AAGCTCGAGGTCTACTTCTGTGACGCCTCCG	EMSA

The restriction enzyme cutting sites are underlined.

## Data Availability

Not applicable.

## Supplementary Materials

The following supporting information can be downloaded at: https://www.mdpi.com/article/10.3390/ijms23052889/s1.
