# Supplementary material for: Expression of the AHPND Toxins PirAvp and PirBvp Is Regulated by Components of the Vibrio parahaemolyticus Quorum Sensing (QS) System
Source: Int J Mol Sci. 2022 Mar 7;23(5):2889. doi: 10.3390/ijms23052889 (PMC8911003; doi:10.3390/ijms23052889)
Supplement: Supplementary file 1 [file ijms-23-02889-s001.zip › ijms-1594421-supplementary.pdf]

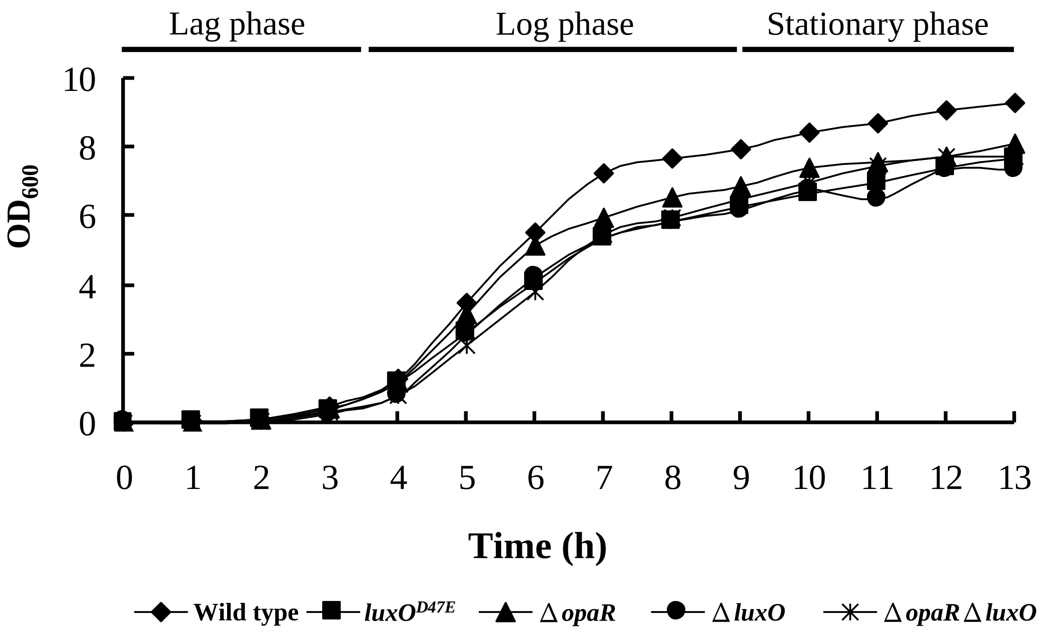

Figure S1. Growth curve of *V. parahaemolyticus* strain 3HP and its mutants (*luxO*^{D47E}, Δ*opaR*, Δ*luxO*, Δ*opaR*Δ*luxO*). Bacteria were cultured in LB+ medium (2% NaCl), and the growth curve was recorded every hour until 13 hours after inoculation. The growth rates were similar for all mutant strains and wild-type strains.
